# Trauma Memory Characteristics and Neurocognitive Performance in Youth Exposed to Single-Event Trauma

**DOI:** 10.1007/s10802-024-01171-3

**Published:** 2024-02-08

**Authors:** Joanna Reed, Richard Meiser-Stedman, Tim Dalgleish, Ben Goodall, Isobel Wright, Adrian Boyle, Aaron Burgess, Fionnuala Murphy, Caitlin Hitchcock, Susanne Schweizer, Emma Travers-Hill, Clare Dixon, Cari-lène Mul, Patrick Smith, Jill Newby, Anna McKinnon

**Affiliations:** 1 Department of Clinical Psychology, University of East Anglia, Norwich, UK; 2 Department of Clinical Psychology and Psychological Therapies, University of East Anglia, Norwich, UK; 3 Medical Research Council, Cognition and Brain Sciences Unit, Cambridge, UK; 4 Cambridge University Hospitals NHS Foundation Trust, Cambridge, UK; 5 Sussex Partnership NHS Foundation Trust, Worthing, UK; 6 Institute of Psychiatry, Psychology and Neuroscience, King’s College London, London, UK; 7 Centre for Emotional Health, Macquarie University, Sydney, Australia

**Keywords:** Trauma memory, Neurocognition, Child, Adolescent

## Abstract

Cognitive models of posttraumatic stress disorder (PTSD) highlight characteristics of trauma memories, such as disorganisation, as key mechanisms in the aetiology of the disorder. However, studies investigating trauma memory in youth have provided inconsistent findings. Research has highlighted that PTSD in youth may be accompanied by difficulties in neurocognitive functioning, potentially impacting ability to recall the trauma memory. The present study sought to investigate both trauma memory characteristics and neurocognitive functioning in youth aged 8–17 years. Youths exposed to single-event trauma, with (*N* = 29, M_age_ = 13.6, 21 female) and without (*N* = 40, M_age_ = 13.3, 21 female) a diagnosis of PTSD, completed self-report measures of trauma memory, a narrative memory task and a set of neurocognitive tests two to six months post-trauma. A group of non trauma-exposed youths (*N* = 36, M_age_ = 13.9, 27 female) were compared on narrative and neurocognitive tasks. Results indicated that trauma memories in youth with, versus without, PTSD were more sensory-laden, temporally disrupted, difficult to verbally access, and formed a more ‘central’ part of their identity. Greater differences were observed for self-reported memory characteristics compared to narrative characteristics. No between group differences in neurocognitive function were observed. Self-reported trauma memory characteristics highlight an important factor in the aetiology of PTSD. The observed lack of significant differences in neurocognitive ability potentially suggests that cognitive factors represent a more relevant treatment target than neurocognitive factors in single-event PTSD. Further research to understand the cognitive factors represented by self-reported trauma memory characteristics is recommended.

**Supplementary Information:**

The online version contains supplementary material available at 10.1007/s10802-024-01171-3.

## Introduction

Exposure to traumatic experiences in childhood and adolescence can result in distressing psychological sequalae in the form of post-traumatic stress disorder (PTSD; American Psychiatric Association, 2013). It is important to understand the processes underpinning the development and maintenance of PTSD in this population to facilitate effective psychological interventions.

Cognitive models of PTSD highlight characteristics of trauma memories, such as fragmentation and disorganisation, as key mechanisms in the development and maintenance of the disorder (Brewin et al., 1996; Ehlers & Clark, 2000). It is proposed that high levels of peritraumatic threat and ‘data-driven’ processing, i.e. processing sensory and perceptual characteristics of the event, as opposed to the meaning of the event (Halligan et al., 2003; McKinnon et al., 2008), impairs the encoding process. This can result in memories of the traumatic event that are not fully integrated into their autobiographical context. These memories are instead fragmented, disorganised, sensory-laden, temporally disrupted and easily triggered by related environmental cues (Brewin et al., 2010; Sündermann et al., 2013), thus giving rise to intrusive reliving symptoms and poorer post-trauma adjustment (Ehlers & Clark, 2000).

However, support for this ‘special mechanisms’ view has not been unanimous. Some authors have argued instead for the ‘basic mechanisms’ view, proposing that reliving symptoms reflect greater availability and repeated rehearsal of trauma memories, due to these memories forming a central part of an individual’s life story (Rubin et al., 2008). It is important to clarify the processes underlying post-traumatic stress symptoms, as trauma-focused cognitive behavioural therapy (TF-CBT), the recommended first-line treatment for PTSD in youth (National Institute for Health and Care Excellence, 2018), bases its key elements on the ‘special mechanisms’ view (Ehlers & Clark, 2000; Kangaslampi & Peltonen, 2019).

Different methodologies are available to investigate trauma memories, including self-report questionnaires such as the Trauma Memory Quality Questionnaire (TMQQ; Meiser-Stedman et al., 2007), or narrative recall of the traumatic event. It could be argued that narrative recall may offer a more detailed means of investigating the distinctive properties of trauma memory proposed by cognitive theory (Crespo & Fernandez-Lansac, 2016). However, this method is not without limitations, as anxiety during recall may activate cognitive avoidance, resulting in sparse narratives that do not reflect the true experience of the trauma (Gray & Lombardo, 2001). There is currently limited literature investigating trauma memory characteristics in youth populations, and that which is available has produced mixed findings. Some studies have observed an association between greater disorganisation of trauma narratives and higher levels of post-traumatic stress symptoms (Kenardy et al., 2007; Salmond et al., 2011), whilst others indicate greater coherence of narratives in children experiencing higher levels of post-traumatic stress symptoms (O’Kearney et al., 2007). McKinnon et al. (2017) found that reduced cohesion and greater negative emotion was associated with acute post-traumatic stress symptoms, however these qualities were not predictive of later post-traumatic stress symptoms. This study also utilised self-report methodology and found scores on the TMQQ to be a greater predictor of post-traumatic stress symptoms than narrative recall characteristics. McGuire et al. (2021) similarly found that self-reported memory characteristics were associated with acute post-traumatic stress symptoms, whereas this association was not observed for narrative memory characteristics. This highlights the importance of combining both self-report and narrative methodology.

In addition to the aforementioned cognitive processes, neurobiological conceptualisations of PTSD highlight a potential role for neurobiological factors in the development of post-traumatic stress symptoms. It is proposed that prolonged activation of the physiological stress response alters brain neurochemistry, with deleterious effects on the function of hippocampal and frontal lobe regions (Yehuda et al., 2015), contributing to re-experiencing symptoms and broader neurocognitive dysfunction. A meta-analysis of neurocognitive function in young people with PTSD has highlighted deficits in general intelligence, language and verbal skills, perceptual and visuospatial skills, and executive function (Malarbi et al., 2017). However, the majority of studies focused on enduring familial trauma, and low socioeconomic status has been identified as a separate risk factor for both familial trauma and poorer cognitive function (Hackman et al., 2015; Paxson & Waldfogel, 2002). Therefore, current conclusions regarding neurocognitive function in young people with PTSD are confounded by the influence of socioeconomic status. TF-CBT typically relies on detailed recollection of the trauma event and the capacity to integrate new learning (Kangaslampi & Peltonen, 2019) and it is plausible that neurocognitive difficulties may detrimentally affect this process (Nijdam et al., 2015). Therefore, investigation of neurocognitive functioning in young people exposed to non-chronic, single-event trauma is warranted to understand whether concerns regarding neurocognitive functioning are relevant to this population.

The current study will be the first to investigate trauma narratives, using self-report questionnaire and narrative recall methodology, and neurocognitive function together in a youth sample exposed to single-event trauma. Firstly, the study aims to investigate trauma memories in trauma-exposed youth, both with and without a diagnosis of PTSD. Secondly, the study aims to explore neurocognitive function in TE youth with that in youth who have not experienced trauma, using a standardised battery of neurocognitive tests. The following research questions are proposed:

1. Do trauma narratives in youth with PTSD significantly differ on trauma memory characteristics, as indexed by self-report questionnaire and narrative recall methodology, compared to trauma-exposed youth without PTSD?
2. Are there significant differences in neurocognitive functioning in youth with PTSD compared to trauma-exposed youth without PTSD and non trauma-exposed youth?

Based on cognitive theory and the ‘special mechanisms’ view, we hypothesised that young people with PTSD would demonstrate higher levels of sensory and negative emotional content, disorganisation, incoherence, and temporal disruption in their trauma narratives compared to trauma-exposed youth without PTSD. Drawing upon the ‘basic mechanisms’ view, we also hypothesised that the trauma memory would be more ‘central’ to identity and life story in youth with, versus without, PTSD. Potentially confounding factors in the neurocognitive literature are not yet definitively understood, therefore we hypothesised that young people with PTSD would demonstrate poorer neurocognitive functioning compared to trauma-exposed youth without PTSD on the basis of currently available evidence. This was considered more exploratory given that the current study is the first to explore neurocognitive function in single-event trauma.

## Method

### Participants

One-hundred-and-five 8–17-year-olds were recruited as parts of the Acute Stress Programme for Children and Teenagers (ASPECTS) study to facilitate a case-control study and subsequent randomised controlled trial (RCT). Trauma-exposed participants were recruited from local emergency departments as part of an earlier screening and prospective longitudinal study to explore the development of PTSD following a traumatic incident. Only a minority of this sample subsequently developed PTSD. Details of recruitment and exclusion criteria of the screening study are available in Meiser-Stedman et al. (2019). The Children’s PTSD Inventory (Saigh et al., 2000) and was used to determine a diagnosis of PTSD two months post-trauma. The measure is a self-report structured interview that has shown good reliability and validity in youth populations (Yasik et al., 2001). Relevant to the current study, a group of 29 participants with PTSD (M_age_ = 13.6, 21 female) were recruited. Ten were recruited from the ASPECTS case-control study and an additional 19 were recruited from community mental health teams, family doctors, schools and adverts in health clinics to participate in the RCT. A control group of 40 trauma-exposed participants that did not meet the diagnostic criteria for PTSD (M_age_ = 13.3, 21 female) were also recruited from the ASPECTS case-control study. The trauma-exposed control group offered the opportunity to establish whether observed results were attributable specifically to PTSD or to trauma exposure more generally. A control group of 36 participants (M_age_ = 13.9, 27 female) without trauma exposure was recruited through schools in the region covering a diverse catchment area and thus with matched socioeconomic background to the trauma-exposed groups. This was important given the potentially confounding role of socioeconomic status in neurocognitive function, as previously described. The current study was limited to the available sample size which was recruited for the primary purpose of facilitating the prospective longitudinal study and RCT. See Table 1 for demographic characteristics and trauma type data.

**Table 1:** Participant demographics

	PTSD (*N* = 29)	Trauma-exposed, Non-PTSD^1^ (*N* = 40)	Non trauma-exposed (*N* = 36)
Age (years)	13.65 (2.5)	13.27 (3.1)	13.91 (2.4)
Female sex	21 (72.4%)	21 (52.5%)	27 (75%)
Minority ethnicity	5 (17.2%)	3 (7.5%)	4 (11.2%)
Household income > 20 K	17 (58.7%)	29 (72.5%)	25 (69.4%)
Trauma type			
RTA	15 (51.7%)	19 (47.5%)	-
Assault	7 (24.1%)	5 (12.5)	-
Accidental injury	3 (10.3%)	14 (35%)	-
Other	4 (13.8%)	-	-

Note: RTA = Road traffic accident. Values presented as Mean (SD) or Frequency (%)

^1^Data on trauma type missing for two participants from trauma-exposed non-PTSD group

### Procedure

Trauma-exposed participants were assessed for PTSD two to six months post-trauma as described in Meiser-Stedman et al. (2019). There was no significant difference in time since trauma between trauma-exposed youth with PTSD and trauma-exposed youth without PTSD, *t* (67) = 0.66, *p* = .95. Participants completed questionnaires on post-traumatic stress symptoms and trauma-relevant psychological processes. All participants completed a narrative task and a battery of neurocognitive tests. For the narrative task, participants were instructed to give a verbal account of their trauma event and a recent negative event. Non trauma-exposed participants narrated a negative event only. The negative event provided a control narrative, to establish whether memory characteristics were generic to memories of negative valence or specific to trauma memories. The neurocognitive tests included measures of intelligence, memory, attention, and executive functioning. Participants meeting the ICD-10 PTSD (World Health Organisation, 1993) criteria were subsequently invited to participate in a 10-week RCT of cognitive therapy for PTSD (details reported in Meiser-Stedman et al., 2017). The study was approved by the UK National Research Ethics Service, Cambridgeshire 1 Research Ethics Committee (10/H0304/11) and registered with the ISRCTN Registry (ISRCTN38352118). Informed assent/consent from the child and their parent/carer was required for participation.

### Measures

#### Self-Report Questionnaires

The Trauma Memory Quality Questionnaire (TMQQ; Meiser-Stedman et al., 2007) was used to assess the sensory quality, temporal context, and verbal accessibility of trauma memories. The Children’s Data Driven Processing Questionnaire (CDDPQ; McKinnon et al., 2008), adapted from the adult Data-Driven Processing Scale (Halligan et al., 2003), was used as a measure of peritraumatic data-driven processing. A youth-adapted version of the Centrality of Event Scale (Berntsen & Rubin, 2006), named the Children’s Centrality of Event Scale (CCES), was used to assess the extent to which the trauma memory formed a ‘central’ reference point for identity and attribution of meaning to other life experiences. The TMQQ and CDDPQ were chosen due to their specificity for youth populations and favourable psychometric properties (Foa et al., 2001; McKinnon et al., 2008; Meiser-Stedman et al., 2007). All showed excellent internal consistency in the present study (α = >0.90). The CCES was developed specifically for the current study, therefore less data is available on the psychometric properties of this adapted measure. However, the adult version of the scale shows good reliability (Berntsen & Rubin, 2006) and Cronbach’s alpha for the current study indicated excellent internal consistency (α = 0.93).

#### Narrative Task

Trauma-exposed participants were asked to provide a verbal account of the trauma event, and a negative event which had occurred within the last three months (see supplementary materials for narrative task instructions). Non trauma-exposed participants were asked to provide a verbal account of a recent negative event only. Following protocols from previous studies (Foa et al., 1995; Halligan et al., 2003; McGuire et al., 2021; Salmond et al., 2011), narratives were transcribed and chunked into utterances. The content of each utterance was coded according to the following characteristics taken from the Foa et al. (1995) coding protocol: repetitions, disorganised thoughts, organised thoughts, sensations, and negative feelings. Repetitions and disorganised thoughts were converted into Z scores and added together, and the Z score of organised thoughts was subtracted from this to give an overall score pertaining to disorganisation (Halligan et al., 2003; Salmond et al., 2011). Sensations and negative feelings were converted into percentages of the total number of utterances to control for the length of each narrative (McGuire et al., 2021; Salmond et al., 2011). Each narrative was given a score between 1 and 10 to reflect overall incoherence of the narrative, with 1 indicating a highly coherent narrative and 10 indicating a highly incoherent narrative (Halligan et al., 2003). The Narrative Coherence Coding Scheme (Reese et al., 2011) proposes that narrative coherence can be further broken down into three dimensions: context, the extent to which the narrative was orientated into place and time; chronology, the extent to which the narrative was narrated in a sequential order; and theme, the extent to which the narrating ‘hung together’ in terms of a clear beginning, middle and end. Each narrative was given a score between zero and three on each of these three dimensions. A score of zero reflected poor context, chronology and theme, whereas three indicated the narrative was well contextualised, followed a chronological order and followed a clear structure, i.e. theme. Coding of the narratives was completed by two blind raters and a third of the narratives were coded by both raters, to assess interrater reliability. There was good agreement between raters for coherence, context and theme (ICC = 0.77–0.84). However, there was only moderate agreement between raters for chronology (intraclass correlation coefficients [ICC] = 0.50), therefore this variable was excluded from subsequent analysis.

#### Neurocognitive Battery

The Wechsler Abbreviated Scale of Intelligence (WASI; Wechsler, 1999) was used to provide an estimation of Full Scale Intelligence Quotient (FSIQ). The California Verbal Learning Test – Children’s version (CVLT-C; Delis et al., 1991) was used to assess encoding, organisation and retrieval of verbal material. A parallel alternate form was developed by co-author AMK for the purposes of retesting. The stories subtest from the Children’s Memory Scale (CMS; Cohen, 1997) was used as a measure of immediate and delayed verbal memory for auditorily presented material. The digit span subtest of the Wechsler Intelligence Scale for Children – Fourth Edition (WISC-IV; Wechsler, 2003) was used as a measure of verbal working memory. The Continuous Visual Memory Test (CVMT; Trahan & Larabee, 1983) was used as a measure of immediate and delayed visual memory. Sustained attention is necessary to encode information into short- and long-term memory stores (Awh et al., 2006), therefore a simple response time task (SRT) was included as a measure of sustained attention. Participants completed 20 trials in which they focused on a fixation point and were instructed to respond as quickly as possible when a target stimulus appeared. Mean reaction time for the 20 trials was calculated. Two versions of the task were developed, both of approximately equal lengths but differing inter-stimulus intervals. The Computerised Multiple Elements Test (CMET; Hynes et al., 2015), a computerised game variation of the Six Elements Test of the Behavioural Assessment of the Dysexecutive Syndrome (Wilson et al., 1997), was used as a measure of executive functioning. The task measures attentional control, self-regulation, and planning and organisational skills. Neurocognitive tests were selected for their reliability and validity across the age range of the sample. The CMET is the only test with limited evidence to support its reliability and validity in youth populations, however imaging research indicates the ability of the test to activate well-established executive networks and it is suggested that the computerised task may offer greater ecological validity compared to classic paradigms (Fuentes-Claramonte et al., 2021).

The order in which participants provided narratives, the version of simple response time task administered and the standard and alternate versions of the California Verbal Learning Test were counterbalanced between participants (see supplementary materials for counterbalancing conditions). Stories from the Children’s Memory Scale stories subtest were counterbalanced by keeping a log and presenting stories alternately as participants entered the study.

### Analysis

All data were analysed cross-sectionally using between-group comparisons. Data were analysed using SPSS (Version 27.0) and R (Version 4.1.2). To check whether there was any overlap between the numerous variables included within the analyses, a correlation matrix was produced (see supplementary materials 1). As expected, some strong correlations were observed between variables within related groups, i.e. within self-report questionnaires, within narrative task variables, and within neurocognitive task variables. However, no strong correlations were observed between variables belonging to different groups (only narrative incoherence was associated with neurocognitive functioning at a level greater than *r* = .4), suggesting that the self-report questionnaires, narrative task variables, and neurocognitive task variables were measuring distinct constructs. Measures of skewness and kurtosis, in addition to significant Shapiro-Wilk results, indicated that the data did not meet the assumptions for parametric analysis. As previously detailed, the sample size available for the current study was constrained by previous recruitment which had primarily been for the purposes of a prospective longitudinal study and RCT. Post-hoc calculations using G*Power (Faul et al., 2007) indicated that the sample size of the trauma-exposed group with PTSD (*N* = 29) and trauma-exposed group without PTSD (*N* = 40) provided 80% power to detect medium-large effect sizes (0.69). To correct for skewness and limited power, the WRS2 package (v1.1-3; Mair & Wilcox, 2020) in R was used to apply robust statistical methods to the pre-intervention data, namely bootstrapping and trimmed means. The bootstrapping function repeatedly resampled the study sample to provide an approximation of estimates that would be observed if the whole population was sampled. The number of resamples was 1000. The top and bottom 20% of scores were trimmed and the mean subsequently calculated from the remaining scores, as this has been shown to produce robust test statistics (Wilcox, 2017). Yuen’s modified t-test for independent trimmed means (Yuen, 1974) was used to compare means between trauma-exposed groups for self-report and trauma narrative data. A robust model, equivalent to a one-way ANOVA (Field & Wilcox, 2017) was used to compare trimmed means across both trauma-exposed groups and the non trauma-exposed group for negative event narratives and neurocognitive data. Robust post-hoc tests were used to compare the difference between trimmed means and provide a *p* value for this difference (ψ̂). The robust methods analyses produce an explanatory effect size, ξ (xi), interpreted as small = 0.01, medium = 0.03, or large = 0.5 (Field & Wilcox, 2017). Bootstrapping was not possible for some variables due to insufficient variation in the data. In such cases, non-parametric results and effect sizes are reported instead. A similar profile of results was observed for non-parametric analyses (see supplementary materials 1).

Each narrative variable and neurocognitive test was compared separately between groups. Given the potentially inflated risk of type I errors, Holm-Bonferroni corrections were applied within groups of related results, i.e. self-report data, trauma and negative narrative data, and neurocognitive data (Holm, 1979). Descriptive statistics are reported for the original data without robust methods applied. Reported *p* values and effect sizes pertain to results obtained using robust methods, unless otherwise stated.

## Results

### Self-Report and Narrative Memory Characteristics

As shown in Table 2, significant differences between trauma-exposed youth with PTSD and trauma-exposed youth without PTSD were observed for all self-report measures, supported by large effect sizes. Trauma-exposed youth with PTSD scored significantly higher than trauma-exposed youth without PTSD on the TMQQ indicating greater sensory content, a sense of ‘nowness’ and difficulties verbally accessing trauma memories. Significantly higher scores on the CCES were also observed for trauma-exposed youth with PTSD compared to trauma-exposed youth without PTSD, indicating that the trauma memory was more ‘central’ to identity and life story in youth with PTSD. Trauma-exposed youth with PTSD also scored significantly higher on the CDDPQ indicating increased data-driven processing at the time of the traumatic event compared to trauma-exposed youth without PTSD.

**Table 2:** Self-report and narrative memory characteristics

Variable	Trauma-exposed, PTSD			Trauma-exposed, Non-PTSD			Non trauma-exposed			Statistical test for group	Effect size (ξ)
	Mdn	IQR	N	Mdn	IQR	N	Mdn	IQR	N		
*Self-reported trauma memory*											
Memory quality (TMQQ)	34	7.50	29	17	6.67	40	-	-	-	Y_t_ = -12.19, *p* = < 0.001*	0.95
Memory centrality (CCES)	2.90	1.21	29	1.29	0.71	39	-	-	-	Y_t_ = -7.77, *p* = < 0.001*	0.88
Data driven processing (CDDPQ)	23.50	9	28	13	11	39	-	-	-	Y_t_ = -5.18, *p* = < 0.001*	0.80
*Trauma event narrative*											
Disorganisation	− 0.25	1.13	27	− 0.24	1.04	38				Y_t_ = -0.37, *p* = .71	0.08
Incoherence	3	3	27	2	2	37	-	-	-	Y_t_ = -1.98, *p* = .04	0.36
Sensations	4.80	5.23	27	2.66		36	-	-	-	Y_t_ =-3.15, *p* = .003*	0.50
Negative feelings	1.43	4.20	27	1.45	3.03	35	-	-	-	Y_t_ = -1.06, *p* = .28	0.21
Context	1	1	27	1	0.50	37	-	-	-	U = 492, *p* = .91	− .01^1^
Theme	1	1	27	2	2	37	-	-	-	U = 287.50, *p* = .002*	.38^1^
*Negative event narrative*											
Disorganisation	− 0.13	1.23	27	− 0.27	1.43	37	− 0.41	1.19	36	F_t_ =1.67, *p* = .19	0.25
Incoherence	4	2	27	3	2	36	3	1.75	36	F_t_ =4.05, *p* = .04	0.39
Sensations	0	0	27	0	0	35	0	0.89	36	H(2) = 1.66, *p* = .44	.05^2^
Negative feelings	2.50	6.25	27	2.91	4.31	36	4.26	6.01	36	F_t_ =0.55, *p* = .55	0.16
Context	1	0	27	1	1	36	1	0	36	H(2) = 0.13, *p* = .94	− .02^2^
Theme	1	1	27	2	1	35	2	1	36	H(2) = 2.60, *p* = .27	.01^2^

Note: CCES = Child Centrality of Events Scale, CDDPQ = Child Data Driven Processing Questionnaire, IQR = interquartile range, Mdn = median, TMQQ = Trauma Memory Quality Questionnaire

*Indicates significance at Holm-Bonferroni corrected alpha level. ^1^ Not possible to compute robust statistics for these variables, Mann-Whitney U test used as non-parametric alternative and effect size, *r*, interpreted as small 0.10, medium 0.25, large ≥ 0.40 (Rosenthal & Rosnow, 1991). ^2^ Not possible to compute robust statistics for these variables, Kruskal-Wallis test used as non-parametric alternative. Effect size, η_p_^2^,Interpreted as small 0.01-0.06, medium 0.06-0.14, large ≥ 0.14 (Tomczak & Tomczak, 2014). ^ab^ Indicates significant post-hoc group differences.

Analysis of trauma event narratives demonstrated that youth with PTSD had a significantly higher percentage of sensory content in their narratives and significantly lower scores for theme, suggesting these narratives had less clear structure, compared to trauma-exposed youth without PTSD. There was a trending effect for incoherence, however this did not reach statistical significance at Holm-Bonferroni corrected alpha level (α = 0.01). There were no significant between group differences for any other trauma narrative characteristics.

Although analysis of negative event narratives demonstrated a trending effect for incoherence, this did not reach statistical significance at the Holm-Bonferroni corrected alpha level (α = 0.008). There were no significant between group differences for any other negative narrative characteristics.

### Neurocognitive Function

As shown in Table 3, there were no significant between group differences for any neurocognitive tests. IQ and sustained attention demonstrated medium effect sizes and all other tests demonstrated small effect sizes. Multiple linear regression indicated that IQ and sustained attention were not statistically significant predictors of either TMQQ scores or CCES scores (see supplementary materials 1 for full results).

**Table 3:** Neurocognitive function

*Neurocognitive test*	Trauma-exposed, PTSD			Trauma-exposed, Non-PTSD			Non trauma-exposed			Statistical test for group	Effect size (ξ)
	Mdn	IQR	N	Mdn	IQR	N	Mdn	IQR	N		
IQ (WISC-IV)	95	16	29	101	17	40	101.50	13	36	F_t_ =3.40, *p* = .05	0.34
Verbal memory (CVLT-C)	48	20.50	29	54	13.50	40	54	18	36	F_t_ =1.07, *p* = .35	0.19
Verbal recall immediate (CMS stories subtest)	8	7	29	10	6	40	10.50	4	36	F_t_ =1.54, *p* = .23	0.26
Verbal recall delayed (CMS stories subtest)	8	7.50	28	10	5.75	40	10.50	4	36	F_t_ =0.62, *p* = .54	0.17
Verbal recognition (CMS stories subtest)	7	6.75	28	11	5.75	40	11	6.50	36	F_t_ =1.65, *p* = .21	0.25
Verbal working memory (Digit Span)	9	4	28	10	2.75	40	10	3	36	F_t_ =1.58, *p* = .22	0.22
Visual memory (CVMT)	10	40	28	30	50	40	30	57	35	F_t_ =0.87, *p* = .42	0.19
Sustained attention (SRT)	415.41	103.82	28	381.25	128.84	39	379.61	101.71	36	F_t_ =2.94, *p* = .06	0.31
Executive function (CMET)	108	82.25	29	128	88.75	40	137	96	36	F_t_ =58, *p* = .58	0.15

Note: CMET = Computerised Multiple Elements Test, CMS = Children’s Memory Scale, CVLT-C = California Verbal Learning Test – Children’s version, CVMT = Continuous Visual Memory Test, IQR = interquartile range, Mdn = median, SRT = Simple response time task, WISC-IV = Wechsler Abbreviated Scale of Intelligence – Fourth Edition

## Discussion

The present study aimed to explore self-report and narrative memory characteristics, in addition to neurocognitive function, before psychological intervention in a sample of youth exposed to single-event trauma. The findings indicate significantly greater data-driven processing, as measured by the CDDPQ, in addition to greater self-reported sensory content, sense of ‘nowness’, and difficulty verbally retrieving trauma memories, as measured by the TMQQ, in trauma-exposed youth with, versus without, PTSD, congruent with mechanisms proposed by cognitive models (Brewin et al., 1996; Ehlers & Clark, 2000). Significantly higher scores on the CCES also suggested that trauma memories formed a more ‘central’ part of identity and life story youth with, versus without, PTSD, in line with the ‘basic mechanisms’ view (Rubin et al., 2008). It is therefore difficult to definitively state whether the data provides greater support for the ‘special mechanisms’ view over the ‘basic mechanisms’ view.

Trauma narratives of youth with, versus without, PTSD were significantly more sensory laden, in congruence with cognitive models, but in contrast to previous research which did not find significant differences in the sensory properties of trauma memories in youth (McGuire et al., 2021; McKinnon et al., 2017; O’Kearney et al., 2007; Salmond et al., 2011). Significantly poorer structure, i.e. theme, was also observed, consistent with research observing temporal disruption in trauma narratives (McKinnon et al., 2017). Differences in sensory content and theme were specific to trauma narratives. No significant differences in negative emotional content was observed in trauma narratives, in contrast to McKinnon et al. (2017). Contrary to our hypothesis, and studies using similar methodology (Salmond et al., 2011), we did not observe significant disorganisation in trauma narratives. However, Salmond et al. (2011) did not observe direct between-group differences in disorganisation, but instead found significant differences between trauma and negative event narratives within the PTSD participant group. The present study did not conduct within groups comparison between narratives. Reviews of adult literature have highlighted that sensory characteristics and disturbed temporal aspects of trauma narratives have been observed more consistently than disorganisation (Crespo & Fernandez-Lansac, 2016; O’Kearney & Perrott, 2006). The present study suggests that this assertion may also be relevant to youth populations.

The discrepancy in the magnitude of the differences observed between TE groups in self-report questionnaire versus narrative recall characteristics of trauma memory is consistent with research demonstrating clear differences in self-reported memory, but not narrative recall characteristics, in youth with PTSD (McGuire et al., 2021). Additionally, research has indicated that the association between narrative characteristics and post-traumatic stress symptoms reduces over time (McKinnon et al., 2017); Salmond et al., (2011) considered narrative characteristics only within the acute period following trauma. Given that the present study explored narrative characteristics only within the post-acute period, this may explain the limited differences observed in narrative characteristics.

Whilst the data tentatively indicates that youth with PTSD performed slightly worse across the neurocognitive tests, there were no statistically significant results and no evidence of medium-large effect sizes, contrary to our hypothesis. Given that the sample in the current study were from similar socioeconomic backgrounds, the lack of statistically significant differences between groups may be explained by the suggestion that neurocognitive deficits in youth exposed to chronic trauma may be better explained by environmental risk factors rather than trauma exposure per se (Danese et al., 2017). Although it is noted that there is not definitive data to support this hypothesis. Additionally, a large prospective study has indicated that poorer neurocognitive functioning may in fact precede PTSD and can be conceptualised as a risk factor for victimisation as opposed to an outcome (Danese et al., 2017). It can also logically be proposed that the hypothesised neurophysiological mechanisms would need to enact their effects over a period of time before significant downstream changes in neurocognitive function are observed. However, youth within the study were diagnosed with PTSD within two to six months of the index trauma event. Therefore, it cannot be assumed that the results observed in the current study would generalise to youth exposed to single-event trauma who have experienced PTSD for a more protracted period of time. Additionally, it is acknowledged that there may have been small-medium effect sizes that the current study was not powered to detect. However, the sample size in the current study is similar to many of the studies detailed within Malarbi et al. (2017) and the majority of these studies also reported large effect sizes. Therefore, despite the modest sample size, the current study indicates that the effect sizes associated with differences in neurocognitive function are not as large in single-event PTSD as those which have previously been found in chronic trauma-exposed populations. There may potentially have been some overlap between questions regarding attention difficulties in PTSD measures and neurocognitive tests. Although, it is noted that PTSD measures rely on self-reported perception of attention difficulties, whereas in could be argued that neurocognitive tests may measure this in a more objective way. There was a trending effect suggesting slightly poorer sustained attention in youth with PTSD, however this was statistically non-significant. Additionally, multiple linear regressions suggested that sustained attention was not a statistically significant predictor of TMQQ and CCES scores. Overall, the current results suggest that differences observed in trauma narrative characteristics were more likely to be underpinned by *cognitive* than *neurocognitive* factors. It would be beneficial for future research to replicate these results and undertake longitudinal research in relation to neurocognitive functioning in single-event trauma.

There are several strengths of the current study. The trauma-exposed control group established whether findings were specifically related to PTSD or broadly related to trauma exposure. The negative event narrative allowed us to understand whether narrative characteristics reflected a general recall style in those with PTSD when recalling negative emotional events, or whether these were specific to trauma memories. Recruiting participants from similar socioeconomic backgrounds reduced the potential for this to confound the results and counterbalancing of the experimental battery instils confidence that order effects did not influence the results.

There are some potential limitations of the present study that need consideration. Trauma-exposed participants within this sample experienced a single-event trauma with no clear antecedents. Whilst there is benefit to this, in allowing us to conclude that observed results were unlikely due to wider psychosocial or environmental factors, this also limits the generalisability of the results. Further research in youth with complex multiple trauma histories, e.g. maltreatment, is necessary to conclude whether a similar profile of results for self-report and narrative trauma memory characteristics would be observed in this population. Furthermore, context and theme had a limited range of scores, potentially making them relatively insensitive measures. Limited variation in scores also meant it was not possible to apply robust statistical methods to these variables. Data was not collected on time since negative event in the non trauma-exposed control group, and it could be proposed that a more recent negative event may elicit a stronger emotional response than a less recent negative event. However, there was no significant difference between trauma-exposed groups in the time since trauma, suggesting that this was unlikely to have affected the narrative differences observed between the trauma-exposed groups. The use of cross-sectional group comparisons meant that it was not possible to comment on the extent to which certain factors were associated with post-traumatic stress symptoms or how this relationship may change over time, which may be interesting for future research to consider. As a broader recommendation, it would be beneficial to reduce heterogeneity of narrative coding schemes, as this may contribute to mixed findings observed across studies (O’Kearney & Perrott, 2006). Advancements in technology could be harnessed for these purposes, such as use of artificial intelligence algorithms to reduce subjectivity and human error.

As elaborated in Meiser-Stedman et al. (2017), and confirmed by a recent network analysis (Mavranezouli et al., 2020), TF-CBT demonstrates efficacy in significantly reducing post-traumatic stress symptoms in youth with PTSD. Cognitive theory suggests this is, in part, due to elaboration and subsequent reintegration of trauma memories. However, as noted by other authors, *perceptions* of trauma memory characteristics may represent a more important factor than narrative memory characteristics themselves (Bray et al., 2018; McGuire et al., 2021; McKinnon et al., 2017). It would be interesting to explore whether this may be related to negative appraisals, a cognitive factor consistently identified as important in the aetiology of PTSD (Gómez de La Cuesta et al., 2019; Mitchell et al., 2017). Negative appraisals related to the trauma event and trauma symptoms could potentially influence perceived intensity of these symptoms, which may impact self-report measures such as the TMQQ. It may be the case that challenging negative *perceptions* of trauma memory characteristics during the narrative exposure elements of treatment may be an important target for psychological interventions. It may be beneficial for future research to explore whether an association between self-reported memory characteristics and post-traumatic stress symptoms is mediated by negative appraisals. This is important, as identifying mechanisms of action can help to refine key elements of psychological treatments to improve their efficacy. Additionally, responses on the TMQQ may be capturing the qualities of flashback memories, a specific form of intrusive memory, whereas narrative recall of the trauma memory is qualitatively different in that it is *voluntarily* recalled. It may be beneficial for future research to clearly differentiate between *voluntary* and *involuntarily* recalled trauma memory.

In conclusion, the current results add to an emerging pattern of results within the field of trauma memory in youth, with mixed findings regarding trauma narratives but more consistent findings regarding self-reported memory characteristics, as measured by the TMQQ (McGuire et al., 2021; McKinnon et al., 2017). A lack of significant findings in neurocognitive function suggests that differences in neurocognitive ability are unlikely to underpin differences in memory characteristics. TMQQ scores highlight an important factor in the aetiology of PTSD, however further research is necessary to elucidate cognitive factors represented by these scores, so that these findings may be translated into clinical practice.

### Electronic Supplementary Material

Below is the link to the electronic supplementary material.

Supplementary Material 1

## Data Availability

The data that support the findings of this study are available from the corresponding author upon reasonable request.
