# Supplementary material for: Trauma Memory Characteristics and Neurocognitive Performance in Youth Exposed to Single-Event Trauma
Source: Res Child Adolesc Psychopathol. 2024 Feb 8;52(6):997–1008. doi: 10.1007/s10802-024-01171-3 (PMC11108885; doi:10.1007/s10802-024-01171-3)
Supplement: Supplementary file 1 — Supplementary Material 1 [file 10802_2024_1171_MOESM1_ESM.docx]

**Supplementary Materials**

## ASPECTS Study Recruitment and Procedure Flow Diagram
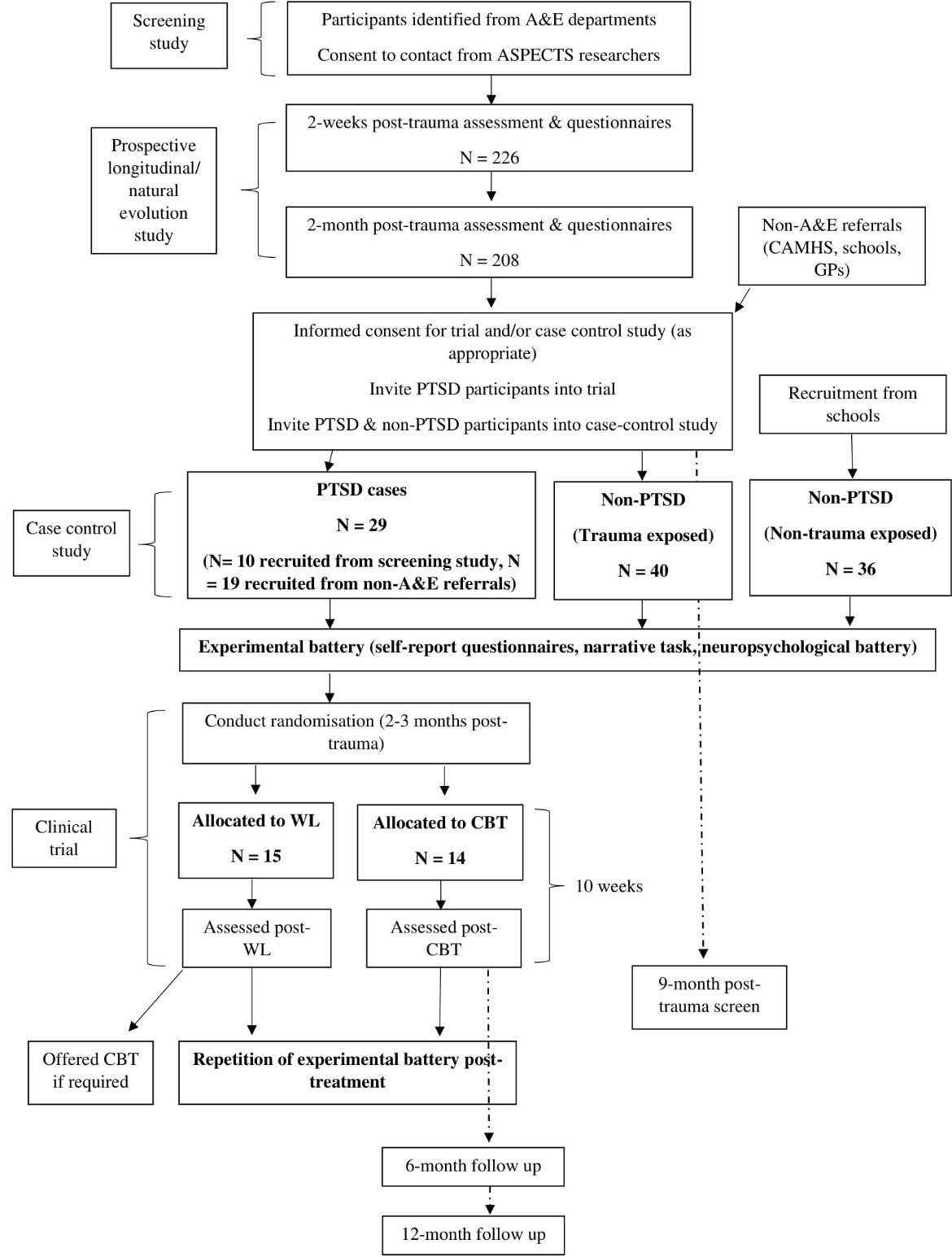


*Note:* Participants and data relevant to the current study are highlighted in bold.

**Narrative task instructions for trauma narrative**

Trauma-exposed participants, with and without PTSD, received the following instructions prior to telling their trauma narratives:

*“Please listen carefully to the following instructions. In a moment I would like you to tell me a story about your [traumatic event] (accident, assault, injury etc). Please tell me about how you felt, what you saw, who was there with you, everything. I would like you to describe this event to me as if it is happening right now. I would like you to tell me as many things that you can remember that happened during the [traumatic event]. Things like what happened around you, how you were feeling, and what you were thinking during the [traumatic event].”*

One standardised prompt was provided after the initial response: *“Can you tell me any more about your frightening event?”*

**Narrative task instructions for negative event narrative**

Trauma-exposed participants, with and without PTSD, and non-trauma exposed participants received the following instructions prior to telling their negative event narratives:

*“Now I would like you to try and think of a negative event that you have experienced in the past three months. This could be anything from getting into trouble at school to having an argument with a friend. The most important thing is that you choose an event which made you feel unhappy, scared, sad, angry, or worried”.*

Trauma-exposed children only also received the instruction, *“This event should not be as scary as your recent trauma”.*

The instructions continued as follows: *“Have you experienced a negative event in the past three months?”*

If a participant had difficulty recalling an event, they were given a calendar and the experimenter discussed different events which had occurred in the past three months.

The instructions continued as follows: *“Please listen to the following instructions carefully. In a moment I would like you to tell me a story about the [negative event], how you felt, what you saw, who was there with you, everything. I would like you to describe the event to me as if it were happening right now. I would like you to tell me as many things that you can remember that happened during the [negative event]. Things like what happened around you, how you were feeling, and what you were thinking during the [negative event].”*

One standardised prompt was provided after the initial response: *“Can you tell me any more about your negative event?”*

## Counterbalancing Conditions for Trauma Exposed and Non-Trauma Exposed Participants

| **Table 1**  *Order of Presentation of Tasks Within Experimental Battery for Trauma-Exposed Participants* | | | | | | | | | |
| --- | --- | --- | --- | --- | --- | --- | --- | --- | --- |
| Condition | Task 1: Narrative | Task 2: Narrative | Task 3 | Task 4 | Task 5 | Task 6 | Task 7 | Task 8 | Task 9 |
| 1 | Trauma | Neg event | CVLT-A | CMS | CVMT | Gamer task | SRT - 1 | WASI | Digit span |
| 2 | Trauma | Neg event | CVLT-S | CMS | CVMT | Gamer task | SRT - 2 | WASI | Digit span |
| 3 | Trauma | Neg event | CVLT-S | CMS | CVMT | Gamer task | SRT - 1 | WASI | Digit span |
| 4 | Trauma | Neg event | CVLT-A | CMS | CVMT | Gamer task | SRT - 2 | WASI | Digit span |
| 5 | Neg event | Trauma | CVLT-A | CMS | CVMT | Gamer task | SRT - 1 | WASI | Digit span |
| 6 | Neg event | Trauma | CVLT-S | CMS | CVMT | Gamer task | SRT - 2 | WASI | Digit span |
| 7 | Neg event | Trauma | CVLT-S | CMS | CVMT | Gamer task | SRT - 2 | WASI | Digit span |
| 8 | Neg event | Trauma | CVLT-A | CMS | CVMT | Gamer task | SRT - 1 | WASI | Digit span |

*Note:* CMS = Children’s Memory Scale, CVLT-A = California Verbal Learning Test - alternate version, CVLT-S = California Verbal Learning Test - standard version, CVMT = Continuous Visual Memory Test, SRT-1 = Simple response time task – version 1, SRT-2 = Simple response time task – version 2, WASI = Wechsler Abbreviated Scale of Intelligence.

| **Table 2**  *Order of Presentation of Tasks Within Experimental Battery for Non Trauma-Exposed Participants* | | | | | | | | |
| --- | --- | --- | --- | --- | --- | --- | --- | --- |
| Condition | Task 1: Narrative | Task 2 | Task 3 | Task 4 | Task 5 | Task 6 | Task 7 | Task 8 |
| 1 | Neg event | CVLT-A | CMS | CVMT | Gamer task | SRT - 1 | WASI | Digit span |
| 2 | Neg event | CVLT-S | CMS | CVMT | Gamer task | SRT - 2 | WASI | Digit span |
| 3 | Neg event | CVLT-S | CMS | CVMT | Gamer task | SRT - 1 | WASI | Digit span |
| 4 | Neg event | CVLT-A | CMS | CVMT | Gamer task | SRT - 2 | WASI | Digit span |

*Note:* CMS = Children’s Memory Scale, CVLT-A = California Verbal Learning Test - alternate version, CVLT-S = California Verbal Learning Test - standard version, CVMT = Continuous Visual Memory Test, SRT-1 = Simple response time task – version 1, SRT-2 = Simple response time task – version 2, WASI = Wechsler Abbreviated Scale of Intelligence.

## Non-Parametric Results for Baseline Memory Characteristics and Neurocognitive function

| **Table 3**  *Non-Parametric Analysis of Self-Report and Narrative Memory Characteristics* | | | | | | | | | | |
| --- | --- | --- | --- | --- | --- | --- | --- | --- | --- | --- |
|  | Trauma-exposed, PTSD | | | Trauma-exposed, Non-PTSD | | | Non trauma-exposed | | |  |
| Variable | Mdn | IQR | N | Mdn | IQR | N | Mdn | IQR | N | Statistical test for group |
| *Self-reported trauma memory* |  |  |  |  |  |  |  |  |  |  |
| Memory quality (TMQQ) | 34 | 7.50 | 29 | 17 | 6.67 | 40 | - | - | - | U= 20.50, *p*=<.001* |
| Memory centrality (CCES) | 2.90 | 1.21 | 29 | 1.29 | .71 | 39 | - | - | - | U= 77.50, *p*=<.001* |
| Data driven processing (CDDPQ) | 23.50 | 9 | 28 | 13 | 11 | 39 | - | - | - | U=149, *p*=<.001* |
| *Trauma event narrative* |  |  |  |  |  |  |  |  |  |  |
| Disorganisation | -.25 | 1.13 | 27 | -.24 | 1.04 | 38 |  |  |  | U= 486.50, *p*=.72 |
| Incoherence | 3 | 3 | 27 | 2 | 2 | 37 | - | - | - | U=379, *p*=.09 |
| Sensations | 4.80 | 5.23 | 27 | 2.66 |  | 36 | - | - | - | U= 289.50, *p*=.006* |
| Negative feelings | 1.43 | 4.20 | 27 | 1.45 | 3.03 | 35 | - | - | - | U=399, *p*=.28 |
| *Negative event narrative* |  |  |  |  |  |  |  |  |  |  |
| Disorganisation | -.13 | 1.23 | 27 | -.27 | 1.43 | 37 | -.41 | 1.19 | 36 | H=4.17, *p*=.13 |
| Incoherence | 4 | 2 | 27 | 3 | 2 | 36 | 3 | 1.75 | 36 | H=5.63, *p*=.06 |
| Sensations | 0 | 0 | 27 | 0 | 0 | 35 | 0 | .89 | 36 | H=1.66, *p*=.44 |
| Negative feelings | 2.50 | 6.25 | 27 | 2.91 | 4.31 | 36 | 4.26 | 6.01 | 36 | H=1.01, *p*=.60 |

*Note:* CCES = Child Centrality of Events Scale, CDDPQ = Child Data Driven Processing Questionnaire, Mdn = median, IQR = interquartile range, PTSD = post-traumatic stress disorder, TMQQ = Trauma Memory Quality Questionnaire.

| **Table 4**  *Non-Parametric Analysis of Neurocognitive Function* | | | | | | | | | | |
| --- | --- | --- | --- | --- | --- | --- | --- | --- | --- | --- |
|  | Trauma-exposed, PTSD | | | Trauma-exposed, Non-PTSD | | | Non trauma-exposed | | |  |
| *Neurocognitive test* | Mdn | IQR | N | Mdn | IQR | N | Mdn | IQR | N | Statistical test for group |
| IQ  (WASI) | 95 | 16 | 29 | 101 | 17 | 40 | 101.5 | 13 | 36 | H=5.61, *p*=.06 |
| Verbal memory  (CVLT-C) | 48 | 20.5 | 29 | 54 | 13.5 | 40 | 54 | 18 | 36 | H=1.62, *p*=.44 |
| Verbal recall immediate (CMS stories subtest) | 8 | 7 | 29 | 10 | 6 | 40 | 10.5 | 4 | 36 | H=3.09, *p*=.21 |
| Verbal recall delayed  (CMS stories subtest) | 8 | 7.5 | 28 | 10 | 5.75 | 40 | 10.5 | 4 | 36 | H=1.65, *p*=.44 |
| Verbal recognition  (CMS stories subtest) | 7 | 6.75 | 28 | 11 | 5.75 | 40 | 11 | 6.5 | 36 | H=3.46, *p*=.18 |
| Verbal working memory (Digit Span) | 9 | 4 | 28 | 10 | 2.75 | 40 | 10 | 3 | 36 | H=2.43, *p*=.30 |
| Visual memory  (CVMT) | 10 | 40 | 28 | 30 | 50 | 40 | 30 | 57 | 35 | H=2.43, *p*=.30 |
| Sustained attention  (SRT) | 415.41 | 103.82 | 28 | 381.25 | 128.84 | 39 | 379.61 | 101.71 | 36 | H=6.97, *p*=.03 |
| Executive function  (CMET) | 108 | 82.25 | 29 | 128 | 88.75 | 40 | 137 | 96 | 36 | H=.48, *p*=.79 |

*Note:* CMET = Computerised Multiple Elements Test, CMS = Children’s Memory Scale, CVLT-C = California Verbal Learning Test – Children’s version, CVMT = Continuous Visual Memory Test, Mdn = median, IQR = interquartile range, SRT = Simple response time task, WASI = Wechsler Abbreviated Scale of Intelligence.

**Multiple Linear Regression Results for TMQQ**

| **Table 6**  *Regression model with baseline TMQQ scores as the criterion variable* | | | | |
| --- | --- | --- | --- | --- |
|  | Unstandardised coefficients | Standardised coefficients | t | *p* |
| Constant | 31.44 |  |  |  |
| Group (PTSD or non-PTSD) | -15.48 | -.85 | -12.71 | <.001 |
| IQ (WASI) | .006 | .008 | .13 | .90 |
| Sustained attention (SRT) | .003 | .06 | .84 | .40 |

*Note:* PTSD = post-traumatic stress disorder, SRT = Simple response time task, TMQQ = Trauma Memory Quality Questionnaire, WASI = Wechsler Abbreviated Scale of Intelligence.

Adjusted R^2^= 72.9 per cent; F= 60.11, *p*<.001

**Multiple Linear Regression Results for CCES**

| **Table 7**  *Regression model with baseline CCES scores as the criterion variable* | | | | |
| --- | --- | --- | --- | --- |
|  | Unstandardised coefficients | Standardised coefficients | t | *p* |
| Constant | 3.42 |  |  |  |
| Group (PTSD or non-PTSD) | -1.29 | -.68 | -7.97 | <.001 |
| IQ (WASI) | -.01 | -.14 | -1.60 | .14 |
| Sustained attention (SRT) | .001 | -.11 | 1.32 | .19 |

*Note:* CCES = Child Centrality of Events Scale, PTSD = post-traumatic stress disorder, SRT = Simple response time task, WASI = Wechsler Abbreviated Scale of Intelligence.

Adjusted R^2^= 56 per cent; F= 28.45, *p*<.001

**Correlation matrix of self-report questionnaires, narrative task measures, and neurocognitive tasks**

|  | Self-report and narrative memory characteristics | | | | | | | | | Neurocognitive functioning | | | | | | | | |
| --- | --- | --- | --- | --- | --- | --- | --- | --- | --- | --- | --- | --- | --- | --- | --- | --- | --- | --- |
|  | TMQQ | CCES | CDDPQ | Disorg. | Incoher. | Sensat. | Negative Feelings | Context | Theme | IQ | Verbal memory | Verbal recall imm. | Verbal recall del. | Verbal recog. | Verbal WM | Visual memory | SA | EF |
| TMQQ | - | .82** | .59** | .12 | .15 | .25* | .10 | .08 | -.30* | -.16 | -.11 | -.26* | -.17 | -.17 | -.04 | -.14 | .25* | -.07 |
| CCES | - | - | .59** | .09 | .20 | .17 | -.03 | -.01 | -.38** | -.28* | -.13 | -.16 | -.04 | -.23 | -.08 | -.22 | .30* | .04 |
| CDDPQ | - | - | - | .10 | .20 | .22 | .02 | .04 | -.38** | -.17 | -.09 | -.13 | -.01 | -.01 | -.06 | -.08 | .05 | .02 |
| Disorg. | - | - | - | - | .45** | 0.14 | .05 | .07 | -.21 | -.21 | .08 | -.23 | -.27* | -.09 | -.21 | .02 | .05 | .08 |
| Incoher. | - | - | - | - | - | .08 | .03 | -.20 | .59** | -.17 | -.26* | -.25* | -.35** | -.26* | -.45** | .01 | .35** | .29* |
| Sensat. | - | - | - | - | - | - | .26* | .11 | -.05 | -.10 | .04 | -.20 | -.13 | -.16 | -.12 | -.11 | -.08 | -.16 |
| Negative feelings | - | - | - | - | - | - | - | -.08 | -.02 | -.03 | -.04 | -.12 | -.14 | -.03 | .02 | -.04 | -.18 | -.04 |
| Context | - | - | - | - | - | - | - | - | .28* | -.16 | .23 | .20 | .23 | -.03 | -.10 | -.19 | -.12 | -.16 |
| Theme | - | - | - | - | - | - | - | - | - | .20 | .19 | .19 | .25* | .13 | .26* | .07 | .36** | -.12 |
| IQ | - | - | - | - | - | - | - | - | - | - | .40** | .49** | .45** | .44** | .35** | .32** | -.24* | -.02 |
| Verbal memory | - | - | - | - | - | - | - | - | - | - | - | .29** | .36** | .39** | .32** | .21* | -.01 | -.04 |
| Verbal recall imm. | - | - | - | - | - | - | - | - | - | - | - | - | .90** | .53** | .19* | -.01 | -.10 | .04 |
| Verbal recall del. | - | - | - | - | - | - | - | - | - | - | - | - | - | .49** | .27** | .06 | -.10 | -.01 |
| Verbal recog. | - | - | - | - | - | - | - | - | - | - | - | - | - | - | .27** | .12 | -.10 | -.19 |
| Verbal WM | - | - | - | - | - | - | - | - | - | - | - | - | - | - | - | .09 | -.21* | -.18 |
| Visual memory | - | - | - | - | - | - | - | - | - | - | - | - | - | - | - | - | .10 | .10 |
| SA | - | - | - | - | - | - | - | - | - | - | - | - | - | - | - | - | - | .22* |

*Note:* CCES = Child Centrality of Events Scale, CDDPQ = Child Data Driven Processing Questionnaire, disorg. = disorganisation, del. = delayed, EF = executive function, imm. = immediate, incoher. = incoherence, recog. = recognition; SA = sustained attention, sensat. = sensations, WM = working memory.

**Correlation is significant at *p*=.01

*Correlation is significant at *p*=.05
